# Changes in hibernating tricolored bat (*Perimyotis subflavus*) roosting behavior in response to white‐nose syndrome

**DOI:** 10.1002/ece3.9045

**Published:** 2022-07-06

**Authors:** Susan C. Loeb, Eric A. Winters

**Affiliations:** ^1^ U.S. Forest Service Southern Research Station Clemson South Carolina USA

**Keywords:** climate change, hibernaculum, microclimate, *Perimyotis subflavus*, tricolored bats, white‐nose syndrome

## Abstract

Understanding animals' behavioral and physiological responses to pathogenic diseases is critical for management and conservation. One such disease, white‐nose syndrome (WNS), has greatly affected bat populations throughout eastern North America leading to significant population declines in several species. Although tricolored bat (*Perimyotis subflavus*) populations have experienced significant declines, little research has been conducted on their responses to the disease, particularly in the southeastern United States. Our objective was to document changes in tricolored bat roost site use after the appearance of WNS in a hibernaculum in the southeastern U.S. and relate these to microsite temperatures, ambient conditions, and population trends. We censused a tricolored bat hibernaculum in northwestern South Carolina, USA, once each year between February 26 and March 2, 2014–2021, and recorded species, section of the tunnel, distance from the entrance, and wall temperature next to each bat. The number of tricolored bats in the hibernaculum dropped by 90.3% during the first 3 years after the arrival of WNS. However, numbers stabilized and slightly increased from 2018 to 2021. Prior to the arrival of WNS, 95.6% of tricolored bats roosted in the back portion of the tunnel that was the warmest. After the arrival of WNS, we observed a significant increase in the proportion of bats using the front, colder portions of the tunnel, particularly during the period of population stabilization and increase. Roost temperatures of bats were also positively associated with February external temperatures. Our results suggest that greater use of the colder sections of the tunnel by tricolored bats could have led to increased survival due to slower growth rates of the fungus that causes WNS in colder temperatures or decreased energetic costs associated with colder hibernation temperatures. Thus, management actions that provide cold hibernacula may be an option for long‐term management of hibernacula, particularly in southern regions.

## Graphical abstract

We censused a tricolored bat hibernaculum in northwestern South Carolina, USA once each year between 26 February and 2 March 2014‐2021 and found a significant change in roosting behavior associated with white‐nose syndrome disease progression. Greater use of colder sections of the hibernaculum after the arrival of white‐nose syndrome may have led to greater survival and consequently, population stabilization and perhaps increase. However, warmer winter temperatures associated with climate change may decrease bats' ability to behaviorally respond to the disease.

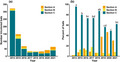

## 1 INTRODUCTION

Over the past several decades, introduced pathogenic diseases such as chytridiomycosis, white‐nose syndrome, and avian malaria have led to significant declines in wildlife populations throughout the world (Carvalho et al., 2017; Cheng et al., 2021; Langwig et al., 2012; Paxton et al., 2016; Scheele et al., 2019). While some species have gone extinct in the face of these diseases, other species have managed to persist, albeit at lower abundance. Persistence in the face of pathogens can be attained through resistance or tolerance where resistance is the ability to limit infection and tolerance is the ability to limit the effects of the pathogen (Brannelly et al., 2021; Råberg et al., 2007; Roy & Kirchner, 2000). Both resistance and tolerance must be considered in the context of the host, pathogen, and environment (i.e., the disease triangle), and both can be achieved through changes in immune function, host behavior, or genetic changes. Knowledge of the mechanisms of resistance and tolerance that allow organisms to persist is critical for guiding management and recovery (Brannelly et al., 2021).

White‐nose syndrome (WNS) is an infectious fungal disease that has killed millions of bats in North America since it was first discovered in New York in 2006 (Hoyt et al., 2021). The fungus that causes WNS, *Pseudogymnoascus destructans*, was most likely introduced from Eurasia (Drees et al., 2017) and affects bats that hibernate in caves, mines, and tunnels (Blehert et al., 2009; Coleman & Reichard, 2014). *P. destructans* is a psychrophilic fungus that grows between 3°C and approximately 19.5°C, with optimal growth occurring between 12.5 and 15.8°C under laboratory conditions (Verant et al., 2012). It also grows best when relative humidity is >70% (Marroquin et al., 2017). To date, 12 bat species have been documented with diagnostic symptoms of WNS (www.whitenosesyndrome.org), but only the little brown bat (*Myotis lucifugus*), northern long‐eared bat (*M. septentrionalis*), Indiana bat (*M. sodalis*), and tricolored bat (*Perimyotis subflavus*) are known to have experienced severe or moderate severity population declines due to the disease while big brown bats (*Eptesicus fuscus*) have suffered serious declines in some areas (Cheng et al., 2021; Langwig et al., 2012; O'Keefe et al., 2019; Powers et al., 2015).

Although populations of little brown bats, northern long‐eared bats, Indiana bats, and tricolored bats have declined significantly throughout much of the eastern U.S., some populations are persisting, though at much lower levels than prior to WNS (Dobony & Johnson, 2018; Langwig et al., 2012; Maslo & Fefferman, 2015). Bats in persisting populations have lower fungal loads than those in declining populations (Langwig et al., 2017) and this resistance may be due to changes in the microbial community on bat wings or an increased immune response (Frick et al., 2017). Greater survival in persisting populations may also be due to greater use of colder and drier sites within or between hibernacula where fungal growth is slower and survival is higher (Hayman et al., 2016; Langwig et al., 2012). For example, little brown bats kept at lower temperatures (4°C) in the laboratory have higher survival than those kept at warmer temperatures (10°C) (Grieneisen et al., 2015; Johnson et al., 2014). In Pennsylvania little brown bats, tricolored bats, and big brown bats roost in sites that are 2–5°C lower than they did prior to the arrival of WNS (Johnson et al., 2016), and little brown bat, big brown bat, and northern long‐eared bat populations increased in colder hibernacula suggesting that roost site selection may be an important strategy for surviving WNS (Turner et al., 2022). Further, fungal loads on little brown bats are lower, and recapture rates are higher at the end of the hibernation period in colder roosts in Midwestern hibernacula although some bats continue to use warmer roosts several years after the appearance of WNS (Hopkins et al., 2021).

Early models of WNS suggested that bats in the southeastern U.S. might experience lower mortality rates than bats in the northeastern U.S. or Canada due to shorter winters (Ehlman et al., 2013). However, warmer temperatures in southern hibernacula resulting in faster fungal growth rates could counter this effect. In the southeastern U.S., temperatures in hibernacula used by tricolored bats are often in the optimal range for *P. destructans* growth (i.e., 12.5–15.8°C) (Lutsch, 2019; Meierhofer et al., 2019; Newman et al., 2021; Sirajuddin, 2018) and the relatively warm hibernacula temperatures in the southeast may explain the high mortality rates observed in this region (>90%) since the arrival of WNS (Pete Pattavina, pers. comm.). Further, the warmer temperatures of these hibernacula may limit bats' ability to use roosts with temperatures well below the optimal growth rate of *P. destructans*.

Historically, the tricolored bat was one of the most common bat species in southern hibernacula (Meierhofer et al., 2019; Perry & Jordan, 2020; Stevens et al., 2017). In addition to hibernating in underground structures such as caves, mines, and tunnels, they also use above‐ground structures such as bridges, culverts, storm drains, water wells, and trees (Ferrara & Leberg, 2005; Fujita & Kunz, 1984; Goering, 1954; Newman et al., 2021; Sandel et al., 2001; Sasse et al., 2011). In northern portions of their range, tricolored bats that hibernate in caves and mines usually select the warmest (7.3–11.8°C) and most humid sites within the hibernaculum (Briggler & Prather, 2003; Kurta & Smith, 2014; Raesly & Gates, 1987), but in Florida, where caves are warmer, they select cooler caves (13.0°C; Smith et al., 2021). Although tricolored bats have experienced significant population declines throughout much of their range, little research on their responses to WNS has been conducted.

Understanding the responses of bats to a variety of environmental conditions across species is critical for predicting bats' responses to WNS and guiding conservation and management actions (Haase et al., 2021). Nonetheless, much of the work on bat responses to WNS have been conducted on little brown bats in the northeastern U.S. Thus, our aim was to determine the responses of tricolored bats to WNS in a hibernaculum in the southeastern U.S. Our specific objective was to document changes in tricolored bat roost positions after the appearance of WNS and relate these to microsite temperatures, ambient conditions, and population trends. The hibernaculum was an unfinished railroad tunnel and had a relatively wide range of temperatures from the more exposed front portion to the more protected back section. Due to the relatively warm temperatures at the back of the tunnel, we predicted that surviving bats would make greater use of the cooler front of the tunnel after the appearance of WNS similar to the movements of bats in northern U.S. hibernacula (Johnson et al., 2016).

## 2 MATERIALS AND METHODS

### 2.1 Study site

The study was conducted in Stumphouse Tunnel in Oconee County, South Carolina. The tunnel was bored through the blue granite mountain side of Stumphouse Mountain and was constructed during the 1850s; however, construction was abandoned in 1859 and consequently, the back end is closed. The tunnel is approximately 493 m long, 5.2 m wide, and 7.6 m high and has three sections (hereafter referred to as Sections A, B, and C), which were separated by brick walls with door‐sized openings. During the study, Section A (the front section) was open to the public, but Sections B and C were not. There was an airshaft approximately halfway through the tunnel in Section B. Mean annual temperature in the area surrounding Stumphouse Tunnel during 2013–2021 was 15.6°C, and mean monthly precipitation was 149.3 mm. Mean daily average winter temperatures (November—March) were 8.4°C with mean average minimum temperatures of 3.8°C and mean maximum temperatures of 13.7°C.

### 2.2 Yearly censuses

We censused Stumphouse Tunnel each year between February 26 and March 2, 2014–2021. Each year, 4–8 people participated in the survey. One or more observers scanned for bats on each side and the ceiling of the tunnel, and one person was designated as the recorder. When a bat was spotted we recorded species, distance from the entrance measured with a Rolatape Model 300 Series measuring wheel (Rolatape), the location (right, left, ceiling), wall temperature next to the bat measured with a Cold Zone infrared thermometer (accuracy of ±0.6°C; Thermoworks, American Fork, UT) or Fluke 62 MAX IR Thermometer (accuracy 1.5%; Fluke Corp.), and section (A, B, or C). If bats were clustered, we recorded the number of bats per cluster. During 2014–2017 we swabbed tricolored bats for the presence of *P. destructans* following previously established protocols (Langwig et al., 2015). Bats were also swabbed in 2013 by the South Carolina Department of Natural Resources. During 2015–2020 we banded any bats that were within arm's reach with Lambourne's 2.4 mm lipped bands. Bats were placed back in their original roosting site. Some bats did not stay in their original roosting site and flew to a new roosting spot. Observers kept track of these bats so that we did not double count bats. Procedures followed the guidelines of the American Society of Mammalogists for the use of wild mammals and were approved by the U.S. Forest Service Institutional Animal Use and Care Committee (AUP #2015–004 and #2021–001). We obtained the mean, minimum, and maximum ambient temperatures for each November through March 2013–2021 from the Remote Automated Weather Station (RAWS) located at the Andrew Pickens Ranger District office, approximately 0.34 km from the tunnel. We also obtained the daily mean, minimum, and maximum ambient temperatures for each February 2014–2021.

### 2.3 Statistical analyses

All analyses were conducted in R 4.0.3 (R Development Core Team, 2020). We used a chi‐square test of independence to test whether the proportion of bats in each section varied with year and used pairwise tests to determine how proportions differed among specific years. Because there were 28 comparisons, we used a Bonferroni correction (α = 0.0018). We conducted a linear regression analysis to determine whether roost wall temperature was related to distance from the entrance and included a year as a random effect. We conducted a one‐way analysis of variance (ANOVA) to test whether winter and February mean, minimum, and maximum temperatures varied among years and a two‐way ANOVA to determine whether roost wall temperatures varied with section, year, and their interaction. We also conducted a two‐way ANOVA to examine the relationship between roost wall temperature and mean February temperatures, section, and their interaction. We used the emmeans package to obtain least‐squared means and conducted pairwise contrasts using the Tukey adjustment (α = 0.05). Least‐squared means ±1 SE are reported.

## 3 RESULTS

**FIGURE 1:**
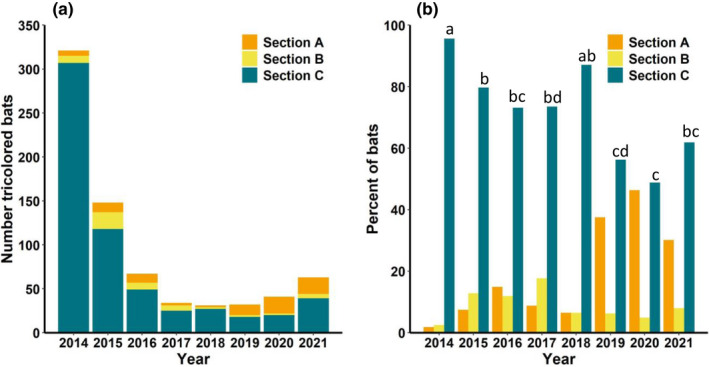
(a) Number of tricolored bats (*Perimyotis subflavus*) in each section of Stumphouse Tunnel, northwestern South Carolina, during yearly censuses conducted during late February or early March 2014–2021 by section, and (b) proportion of tricolored bats in each section. Years with the same letters do not differ significantly in the proportion of bats roosting in each section

The number of tricolored bats in the tunnel ranged from 31 in 2018 (post‐WNS) to 321 (pre‐WNS) in 2014. Tricolored bats made up 96.5% of the bats observed over the 8 years. In addition to tricolored bats, we also counted seven big brown bats (one in 2014, four in 2016, and two in 2021), seven unidentified *Myotis* spp. (three in 2014, two in 2021, and one each in 2015, 2016, 2019, and 2020), and 11 Rafinesque's big‐eared bats (*Corynorhinus rafinesquii*) in 2017. Big brown bats, *Myotis* spp., and Rafinesque's big‐eared bats were only found in Sections A and B. Almost all tricolored bats roosted solitarily, particularly once WNS became evident. We found four clusters of two bats and one cluster of three bats in 2014, one cluster of two tricolored bats in 2015, and no clusters during 2016–2021. None of the 21 tricolored bats swabbed for the presence of *P. destructans* tested positive for the fungus in 2013, but 10 of the 20 bats (50%) swabbed in 2014, nine of 10 bats (90%) swabbed in 2015, eight of 10 (80%) swabbed in 2016, and seven of seven (100%) in 2017 tested positive for the presence of the fungus. We did not observe any signs of WNS (white on the muzzle or wings, dead bats) during 2014, but in 2015, we observed many dead bats on the walls and on the ground. Three of the dead specimens found in 2015 were submitted to the Southeastern Cooperative Wildlife Disease Study and all tested positive for the presence of *P. destructans* and histological examination showed that they had fungal morphology and skin invasion that were diagnostic of WNS. The number of tricolored bats declined by approximately 50% each year from 2014 through 2017 before leveling off through 2019 with slight increases in 2020 and 2021 (Figure 1a). The overall decline between 2014 (pre‐WNS) and 2018 (the lowest point) was 90.3%.

The proportion of bats in each section varied significantly among years (χ^2^ = 158.6, df = 14, *p* < .0001). In 2014, almost all (95.6%) bats were in Section C. During 2015–2017, the proportion of bats in Section C declined significantly with proportionately more bats in Section A and Section B (Figure 1b). In 2019, the proportion of bats occupying Section A increased significantly from previous years and by 2020, almost half the bats were in Section A (Figure 1b). Further, the total number of bats using Section A was higher every year post‐WNS than that in pre‐WNS except 2017 and 2018 when the population was at its lowest points (Figure 1a). For example, in 2014 only six tricolored bats used Section A, but in 2015 and 2016, 10 and 11 tricolored bats were in Section A, respectively, and in 2019 and 2020, 12 and 19 tricolored bats were in Section A, respectively. The number of bats using Section B also increased from 8 in 2014 to 19 in 2015 although the numbers in Section B declined in subsequent years. Nineteen tricolored bats used Section A in 2021; while this number was high compared with pre‐WNS, the proportion was somewhat lower than 2019 and 2020 due to the increase in the total number of bats in the tunnel. Nonetheless, the proportion of bats in Section A was higher than pre‐WNS (2014).

**FIGURE 2:**
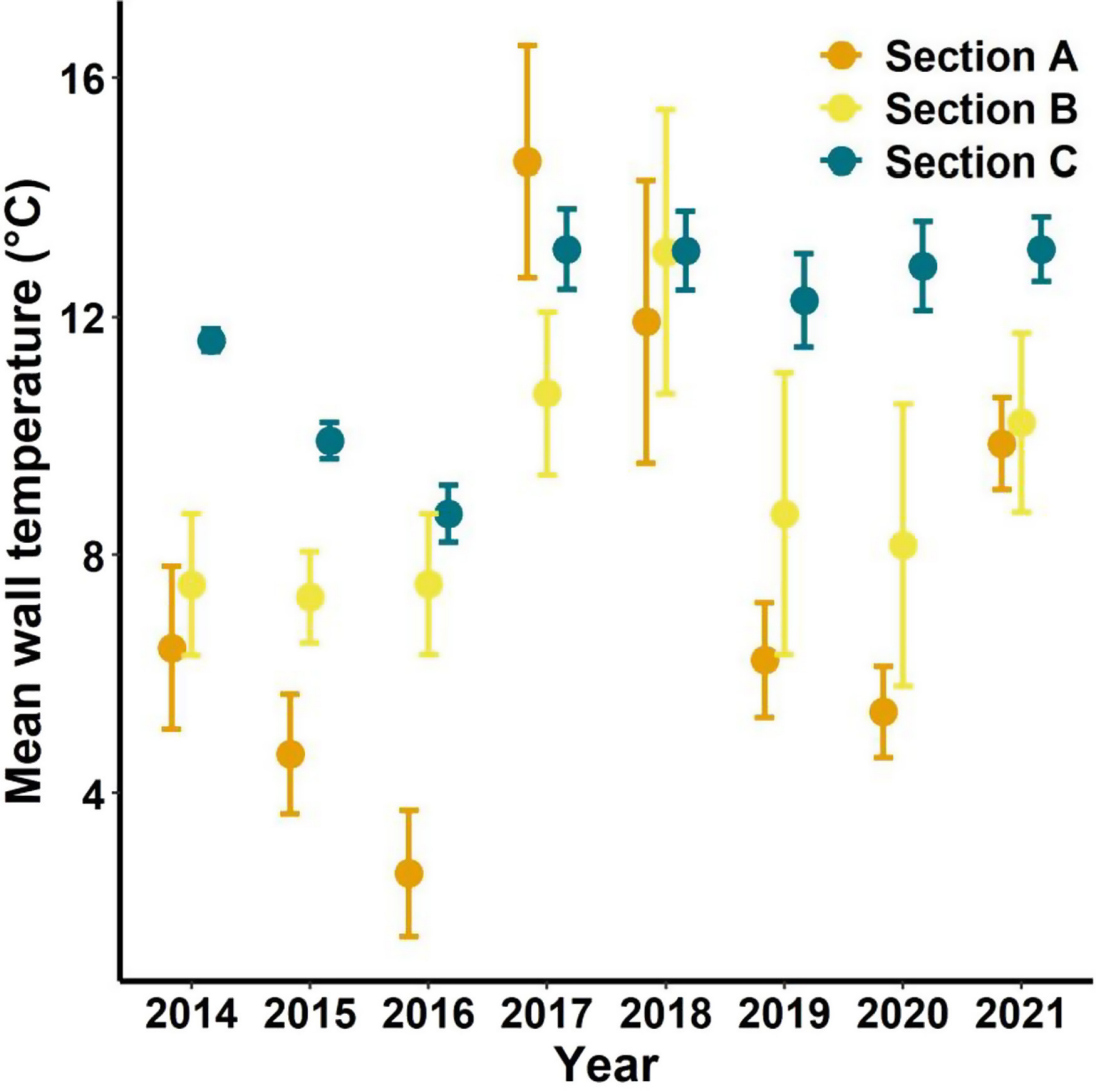
Least‐squared mean ±1 SE of wall temperatures next to hibernating bats by section of Stumphouse Tunnel, northwestern South Carolina, during yearly censuses conducted in late February or early March 2014–2021

Wall temperatures adjacent to bats increased significantly with distance from the entrance (*F*_1,728_ = 1827, *p* < .0001), and varied significantly among sections (*F*_2,712_ = 778.0, *p*  < .0001), years (*F*_7,712_ = 175.6, *p* < .0001), and the interaction (*F*_14,712_ = 18.5, *p* < .0001). For all years combined, mean wall temperatures were significantly lower (*p*  < .05) in Section A (7.71 ± 0.15°C) than in Section B (9.15 ± 0.20°C) and Section C (11.84 ± 0.07°C); the difference between wall temperatures next to bats also differed significantly (*p*  < .05) between Sections B and C. However, there was considerable year‐to‐year variation in wall temperatures within sections, particularly in Sections A and B (Figure 2). Nonetheless, wall temperatures used by bats were significantly lower in Section A than in Section C in every year except for 2017 and 2018 (Figure 2).

**FIGURE 3:**
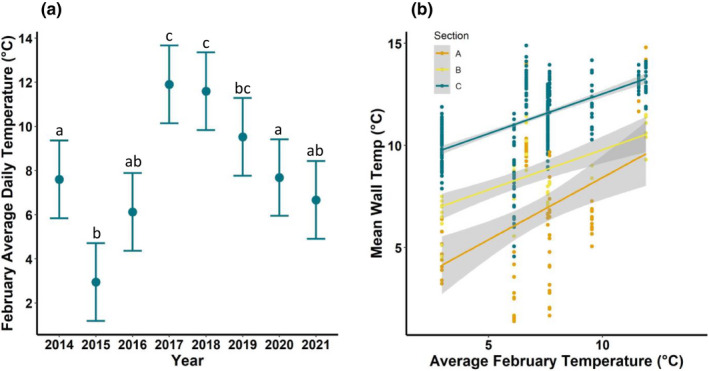
(a) Least‐squared mean daily ambient temperatures during February 2014–2021 in Mountain Rest, South Carolina. Mean values with the same letter do not differ significantly from each other (Tukey's HSD, *p* < .05). (b) Relationship between wall temperature of roosting sites of *Perimyotis subflavus* in each section of Stumphouse Tunnel and average February temperature

Although daily mean, minimum, and maximum temperatures across winters did not vary across years (*F*_7,32_ = 0.74, *p* = .64; *F*_*7,32*_ = 0.91, *p* = .51; *F*_*7,32*_ = 0.57, *p* = .77), daily mean, minimum, and maximum temperatures in February varied across years (*F*_7,217_ = 11.01, *p* < .0001; *F*_7,217_ = 10.04 *p* < .0001; *F*_7,217_ = 8.54, *p* < .0001). Mean daily temperatures during February were significantly warmer during 2017 and 2018 than in every other year except 2019 (Figure 3a). Further, wall temperatures next to bats were positively related to February mean temperatures (*F*_1,730_ = 279.25, *p* < .0001), section (*F*_2,730_ = 359.8, *p* <  .0001), and the interaction (*F*_2,730_ = 3.30, *p* = .04). The slope between wall temperature and February mean temperature was significantly steeper in Section A than in Section B (*p* = .05) and Section C (*p* = .01; Figure 3b).

## 4 DISCUSSION

In contrast to predictions that the effects of WNS would be lower in more southern latitudes (Ehlman et al., 2013), our data indicate that tricolored bats can experience precipitous declines in population numbers after the introduction of *P. destructans* in underground hibernacula in the southeastern U.S. The population decline observed in Stumphouse Tunnel was comparable to declines of tricolored bats, northern long‐eared bats, little brown bats, and Indiana bats in the northeastern U.S. (Frick et al., 2010, 2017; Langwig et al., 2012). Population declines began the year after the first detection of *P. destructans* in the tunnel with numbers dropping by approximately 50% per year for several years and then leveling off between 2017 and 2019, similar to populations of tricolored bats in other parts of the Southern Appalachians (Frick et al., 2017). We observed stabilization and a small increase in the number of bats in the tunnel from 2018 to 2021, and this trend was associated with an increase in the proportion and total number of bats using the coldest parts of the tunnel, similar to observations in the northeastern and Midwestern portions of the U.S. (Hopkins et al., 2021; Johnson et al., 2016; Turner et al., 2022). The increase in the proportion and absolute number of bats in the colder sections of the tunnel after the arrival of WNS may have been due to behavioral changes (i.e., movement to colder parts of the hibernacula), greater survival of bats that roosted in the colder sections, or both. However, the increase in the absolute number of bats using the colder sections after the arrival of WNS (almost doubling in the first few years despite heavy mortality) suggests that at least some of the increase was due to behavioral changes.

The high mortality rates that we observed in the tricolored bat population in Stumphouse Tunnel, despite the shorter and more mild winters experienced by these bats compared with tricolored bats in more northern populations, may have been due to the relatively warm temperatures in the tunnel, particularly Section C where the majority of bats roosted during the years of severe declines (2015–2017). Wall temperatures in Section C in February averaged 11.7°C (this study) and air temperatures in Sections B and C averaged 12.6°C (Sirajuddin, 2018). The optimal growth range of *P. destructans* in the laboratory is 12.5–15.8°C (Verant et al., 2012), and late‐winter *P. destructans* loads are positively correlated with roosting temperature (Langwig et al., 2016). Thus, warmer hibernacula temperatures such as those observed in this study and those observed in Florida (Smith et al., 2021) and in Texas (Leivers et al., 2019; Meierhofer et al., 2019) suggest that bats that hibernate in underground structures throughout the South may be highly susceptible to WNS.

We observed a significant change in the relative distribution of bats across the tunnel after the invasion of WNS. Except for 2018, bats were more likely to roost in the colder sections of the tunnel, particularly Section A, which had the coldest temperatures, after WNS became established. Greater use of colder parts of the hibernaculum, particularly Section A where mean roost wall temperatures in February averaged 7.7°C, may have been due to two reasons. Little brown bats and tricolored bats in poorer conditions (e.g., lower body mass) select colder microclimates than those in better conditions, presumably to reduce energy expenditures (Boyles et al., 2007, 2022). Thus, tricolored bats in Stumphouse Tunnel affected by WNS may have either sought out the colder temperatures of Section A to reduce their energy expenditures, or bats using this section may have had greater survival due to lower energy expenditure. The movement to the colder part of the tunnel may have also resulted in reduced *P. destructans* loads in tricolored bats in Stumphouse resulting in lower disease severity and increased survival and persistence (Langwig et al., 2017; Martinková et al., 2018). For example, population declines of little brown bats in the northeastern U.S. were lower in colder hibernacula in the first few years after the onset of WNS than in warmer hibernacula (Langwig et al., 2012), and little brown bats in the Midwestern U.S. that roost at warmer temperatures have higher fungal loads and lower recapture rates than little brown bats that roost at cooler temperatures (Hopkins et al., 2021). Populations of tricolored bats in Pennsylvania hit their nadir in 2012, after which positive growth only occurred in warmer hibernacula (Turner et al., 2022). However, the temperatures in warmer hibernacula where populations increased in Pennsylvania were comparable to those in Section A of Stumphouse Tunnel. Because tricolored bats rarely form large clusters in hibernacula and often roost solitarily (Fujita & Kunz, 1984) they may need to roost at warmer temperatures than *Myotis* spp., which usually roost in large clusters (Boyles et al., 2008).

Although we saw a significant shift in use from warmer to colder sections of the hibernacula after the arrival of WNS, most bats continued to use the warmest section as they did prior to the arrival of WNS (Figure 1). Similarly, 52% of the little brown bats in Midwestern hibernacula continue to use warmer roosts after the invasion of WNS despite greater survival and lower fungal loads in colder areas (Hopkins et al., 2021). In general, tricolored bats select warmer temperatures than other species of hibernating bats in the eastern U.S. (Briggler & Prather, 2003; Kurta & Smith, 2014; Raesly & Gates, 1987) and when given the choice of hibernation roost temperatures in the laboratory, tricolored bats prefer to roost at 11°C than at 5° or 8°C (Boyles et al., 2022). But, many factors other than thermal/energetic factors contribute to optimal hibernation conditions including nonenergetic physiological costs such as evaporative water loss and waste build‐up, freezing and predation risk, and missed opportunity costs of euthermy such as mating and grooming (Boyles et al., 2020). Thus, Section C may represent optimal hibernation conditions for these bats despite the higher energetic costs of hibernating at warmer temperatures (Humphries et al., 2006). Prior to and after the arrival of WNS, bats may have preferred to roost in Section C due to disturbance from human visitation in Section A and increased predation risks near the entrance (Cichocki et al., 2021; Flaspoler, 2017). For example, bats in Section A are less likely to roost below 2.5 m than those in Section C (R. Brown, unpublished data), which could be due to either disturbance or predation risk (Kokurewicz, 2004). Temperature and humidity were also likely to be more variable in Section A than in Section C (McClure et al., 2020; Vanderwolf & McAlpine, 2021), and thus, roosting in Section A may increase the risk of freezing or result in passive rewarming if temperatures increase (Boyles et al., 2008). During 2017 and 2018 roost temperatures in Section A were not significantly different from those in Section C (Figure 3) and in 2018, the proportion of bats roosting in Section C did not differ from pre‐WNS. This suggests that when there are no thermal or disease‐related advantages to roosting in the riskier site (Section A), bats preferred the more protected Section C.

We observed a positive relationship between February ambient temperatures and roost temperatures, and this relationship was more apparent in Section A than in Sections B and C. If the use of colder temperatures within hibernacula leads to greater survival from WNS (Hopkins et al., 2021; Langwig et al., 2017; Martinková et al., 2018), then warmer February temperatures may limit tricolored bats' abilities to counter the disease in Stumphouse Tunnel and other similar hibernacula. The use of cold microclimates within hibernacula may be particularly important for bats in poor body conditions as roosting at colder temperatures allows bats to have longer and deeper torpor bouts and thus, increase energy savings (Boyles et al., 2007). Many of the hibernacula used by tricolored bats throughout the southeast and Midwest are relatively short. For example, caves in Iowa are rarely longer than 50 m and tricolored bats used 68% of the caves <50 m in length that were surveyed (Dixon, 2011); caves used by tricolored bats in northwestern Arkansas are mostly <200 m (Briggler & Prather, 2003). As temperatures are more variable and reflective of outside temperatures closer to the entrance (Perry, 2013), tricolored bats may be particularly vulnerable to warming temperatures associated with changing climates. Climate change has been identified as a significant threat to bats throughout the world (Frick et al., 2020; O'Shea et al., 2016; Sherwin et al., 2013), and our data suggest that warmer winter temperatures may reduce bats' ability to survive WNS.

Our results suggest that the use of cold roosts may lead to increased survival of tricolored bats in southeastern U.S. hibernacula as the increase in the use of colder roosts was associated with population increases. Although bats continued to use warm roosts, over time it is likely that survival will be lower for those bats as has been observed for little brown bats (Hopkins et al., 2021). Thus, management actions that maintain or even lower temperatures in hibernacula may be an option for improving the survival of bats in areas with WNS (Hopkins et al., 2021; Johnson et al., 2016; Richter et al., 1993), particularly in southern regions where options for colder roosts may be more limited. For example, Turner et al. (2022) modified mines, caves, and an abandoned railroad tunnel in Pennsylvania to lower their temperatures resulting in greater use of these areas by little brown bats, big brown bats, small‐footed bats (*M. leibii*), and northern long‐eared bats. Factors that affect bats' use of colder areas, such as disturbance from humans or predation risk, should also be considered and mitigated where possible. Understanding the energetic, ecological, and environmental factors that affect bats' roosting preferences during hibernation will be critical for effective management, conservation, and recovery of bat populations that have been impacted by WNS.

## AUTHOR CONTRIBUTIONS

**Susan C Loeb:** Conceptualization (lead); data curation (equal); formal analysis (lead); investigation (equal); methodology (equal); writing – original draft (lead); writing – review and editing (lead). **Eric A. Winters:** Data curation (equal); investigation (equal); methodology (lead); writing – original draft (supporting); writing – review and editing (supporting).

## Data Availability

The data that support the findings of this study will be openly available at Dryad www.datadryad/stash.
